# Longitudinal Investigation of the Gut Microbiota in Goat Kids from Birth to Postweaning

**DOI:** 10.3390/microorganisms8081111

**Published:** 2020-07-24

**Authors:** Yimin Zhuang, Jianmin Chai, Kai Cui, Yanliang Bi, Qiyu Diao, Wenqin Huang, Hunter Usdrowski, Naifeng Zhang

**Affiliations:** 1 Feed Research Institute, Chinese Academy of Agricultural Sciences, Key Laboratory of Feed Biotechnology of the Ministry of Agriculture, Beijing 100081, China; zym1164323345@163.com (Y.Z.); jchai@uark.edu (J.C.); cuikai@caas.cn (K.C.); vetbi2008@163.com (Y.B.); diaoqiyu@caas.cn (Q.D.); m13121271017@163.com (W.H.); 2 Department of Animal Science, Division of Agriculture, University of Arkansas, Fayetteville, AR 72701, USA; hmusdrow@uark.edu

**Keywords:** gut microbiota, goat, ruminants, ages, biogeography, temporal dynamics

## Abstract

Early microbial colonization in the gut impacts animal performance and lifelong health. However, research on gut microbial colonization and development in young ruminants, especially after weaning, is currently limited. In this study, next-generation sequencing technology was performed to investigate the temporal dynamic changes of the microbial community in the jejunum and colon of goats at 1, 7, 14, 28, 42, 56, 70, and 84 days (d) of age. As age increased, significant increases in microbial diversity, including the number of Observed OTUs and the Shannon Index, were observed in both the jejunum and colon. Regarding beta diversity, significant shifts in community membership and structure from d1 to d84 were observed based on both Bray–Curtis and Jaccard distances. With increasing age, dominant genera in the jejunum shifted from *Lactobacillus* to unclassified *Ruminococcaceae*, unclassified *Lachnospiraceae* and unclassified *Clostridiales* through starter supplementation, whereas colonic dominant genera changed from *Lactobacillus* and *Butyricicoccus*, within d1–d28, to unclassified *Ruminococcaceae*, unclassified *Clostridiales* and *Campylobacter* after solid diet supplementation. The linear discriminant analysis (LDA) effect size (LEfSe) analysis revealed bacterial features that are stage-specific in the jejunum and colon, respectively. In the jejunum and colon, a significantly distinct structure and membership of the microbiota was observed across all ages. The growth stage-associated microbiota in each gut compartment was also identified as a marker for biogeography. Our data indicate the temporal and spatial differences of the gut microbiota in goats are important for their performance and health. Early microbial colonization can influence microbial composition in later life (e.g., post-weaning phase). This study provides insights that the temporal dynamics of gut microbiota development from newborn to post-weaning can aid in developing feeding strategies to improve goat health and production.

## 1. Introduction

With next-generation sequencing development, the gut microbiota, which plays important roles in nutrient digestion, health, and disease of animals, can now be more deeply investigated. Complex microbial communities quickly colonize the gastrointestinal tract of neonatal animals and mature with age. Early colonization of the gut microbiota is known to impact animal performance and lifelong health [1,2]. As age increases, the factors including anatomical developments, gut physiological environment, diet changes, weaning and administration of antibiotics can generate collateral effects that can influence gut microbiota development and may lead to microbial dysbiosis and subsequent diarrhea or other diseases [3,4,5,6]. Regarding newborn ruminants, gut function develops differently to other species [6,7]. When the rumen is not sufficiently developed and does not yet have ruminant function, digestion patterns of neonate kids are mostly dependent on the small and large intestines [8], and are more similar to those of monogastric animals. During the development of the rumen from the nonfunctional phase to the ruminant phase, the major digestive compartment moves from the intestine to the rumen since the metabolites shift from glucose to volatile fatty acids [7]. Simultaneously, with increasing age, the small intestine develops in response to the alteration of enzymatic activity from lactase to maltase [9]. Therefore, the intestine of ruminants plays important roles in early life, and its microbiota may differ from other mammals. Understanding the longitudinal changes of the intestinal microbiota allows us to better identify the roles of the microbiota in nutrient digestion and isolate probiotics.

To date, there are numerous studies that have investigated the rumen microbiota in ruminants [6,10]. Limited studies have determined the temporal changes of microbiota in the small and large intestines of newborn ruminants, especially gut microbiota after weaning. Li and their colleagues found that an increasing trend of microbial diversity and richness in the duodenum, jejunum, ileum, cecum, and colon with age (0, 14, 28, 42, and 56 days old) was observed in goat kids preweaning [7]. Dias et al. estimated the bacterial development of the rumen, jejunum, cecum, and colon in dairy claves during preweaning development [9]. Another study investigated the ileal microbiota of goat at 0, 7, 28, 42, and 70 days [11].

Although these studies expended our knowledges of gut microbiota in ruminants, a lack of intestinal microbial changes postweaning and comparison of spatial gut microbiota influences deep understanding of the gut ecosystem. Therefore, it is necessary to systematically investigate the temporal dynamic of intestinal microbial development in ruminants. In this study, next-generation sequencing technology was performed to investigate the temporal dynamic changes of the microbial community in the goats’ jejunum and colon at 1, 7, 14, 28, 42, 56, 70, 84 days of age. The microbial dissimilarities between the small and large intestines were also determined. This work prompts us to further understand the developmental rules of intestinal microbes in young ruminants, and provides insights into improving animal feeding strategy, health, and production.

## 2. Materials and Methods

### 2.1. Animal Trial and Sample Collection

**Figure 1:**
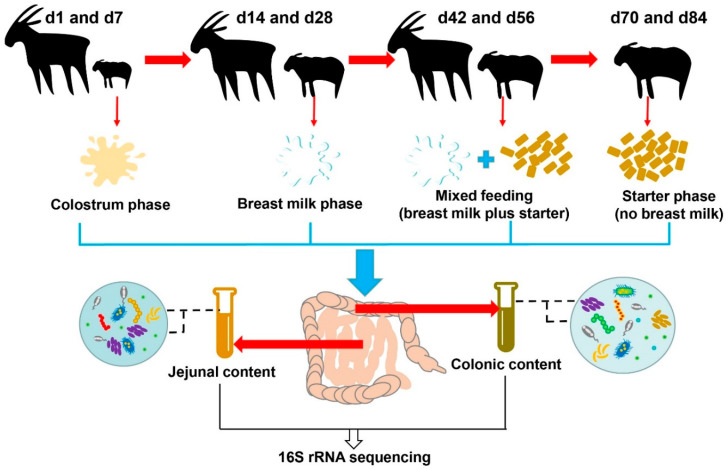
Experimental design and workflow.

The study was conducted in accordance with the guidance of the Animal Ethics Committee of the Chinese Academy of Agricultural Sciences (Protocol Number: AEC-CAAS-20191105; Approval date: 3 November 2019). A total of 48 healthy female Laiwu black goats were selected for this experiment in a goat farm (Laiwu, Shandong province, China). Regarding the age and diets, eight ages (1, 7, 14, 28, 42, 56, 70, 84 days (d) of age) were selected and each age had six replicates (Figure 1). Goat kids lived with their dams and consumed breast milk as the only food source for the first 30 days, which included the colostrum phase (d1 and d7) and breast milk phase (d14 and d28). Goats were supplemented a solid concentrate (nutrient level in Table S1) starting on d 30, and were weaned off breast milk at d 60. Solid concentrate starter was the only food source for the goat kids after d 60. Based on this feeding strategy, we defined the ages after starter supplementation into the mixed feeding phase (breast milk supplemented with starter; d42 and d56) and the starter feeding phase (d70 and d84). During the trial, all goat kids had ad libitum access to water and concentrate. On d1, newborn kids were selected after they suckled colostrum. All goat kids were slaughtered on the respective deadline to collect the contents of the jejunum and colon for microbial analysis. Finally, the collected samples were snap-frozen in liquid nitrogen and subsequently stored in a freezer at −80 °C until sequencing analysis.

### 2.2. DNA Extraction and 16S rRNA Sequencing

A DNeasy PowerSoil Kit (Cat. No. 12888, Qiagen, Valencia, CA, USA) was used to extract microbial DNA from samples of the jejunum and colon. Total DNA quality was checked using a Thermo NanoDrop 2000 UV microphotometer and 1% agarose gel electrophoresis. The V3–V4 region was amplified using adaptor-linked universal primers (341F: CCTACGGGRSGCAGCAG; 806R GGACTACVVGGGTATCTAATC). The diluted genomic DNA was used as a template, and PCR was performed using a high-fidelity enzyme in the KAPA HiFi Hotstart ReadyMix PCR kit to ensure the accuracy and efficiency of the amplification. The AxyPrep DNA Gel Recovery kit (AXYGEN Inc., Union city, CA, USA) was employed to cut the gel and recover the PCR products. To prevent contamination from reagents, negative controls for DNA extraction and PCR amplification were included, and no PCR products of the negative controls were detected from agarose gel. Library quality was checked using a Thermo NanoDrop 2000 UV microphotometer and 2% agarose gel electrophoresis. A Qubit 2.0 Fluorometer (Thermo Fisher Scientific, Waltham, MA, USA) was used to quantify the library. Amplicon libraries were sequenced using an Illumina Miseq PE250 platform (Realbio Technology Genomics Institute, Shanghai, China).

### 2.3. Microbial Data Analysis

The mothur software (version 1.39.1, available online: https://mothur.org/wiki/miseq_sop/mothur) [12] was used to process the raw sequences. Based on the MiSeq SOP, quality-filtering steps, alignment to the SILVA (v132) database, and clustering into operational taxonomic units (OTUs) at the level of 97% similarity was processed. High quality reads were classified against the RDP (Ribosomal Database Project) database [13]. Alpha diversity (Shannon Index and Observed OTUs) of the two groups was compared using a two-tailed Wilcoxon signed-rank test. Beta diversity based on Bray–Curtis and Jaccard distances was tested using an analysis of similarity (ANOSIM). The outputs of diversity were visualized using the “ggplot2” package in R (version 3.6.0, available online: https://www.r-project.org/). The microbial sequencing dates of this study are available in the NCBI SRA database with the BioProject ID PRJNA628143.

The linear discriminant analysis (LDA) effect size (LEfSe), an analytical tool for discovering and interpreting biomarkers of high-dimensional data, was used to identify the signature bacteria associated with the growth stages and intestinal segments. LDA score > 2 was used as a criterion for judging the significant effect size [14]. The signature bacteria were visualized in a heat map using the ‘pheatmap’ function in R.

Network analysis for the intestinal microbiota was performed using the FAST LSA software (version 1.0, available online: http://hallam.microbiology.ubc.ca/fastLSA/install/), which expands the boundaries of LSA analysis on datasets with multiple co-varying time series [15]. The input parameters were set as: maximum time lag 5, minimum LSA value 0.6 and the significance level as 0.05.

The Random Forest regression model was performed to identify the gut bacteria that were related to growth performance (body weight). The ‘randomForest’ package (version 4.6-14, available online: https://cran.r-project.org/web/packages/randomForest/) in R was used. The parameters of ‘importance’ and ‘proximity’ were set as ‘True’, and the ‘ntree’ was set to 10,000 trees.

## 3. Results

### 3.1. The Gut Microbiota Differentiates with the Age of Goats

**Figure 2:**
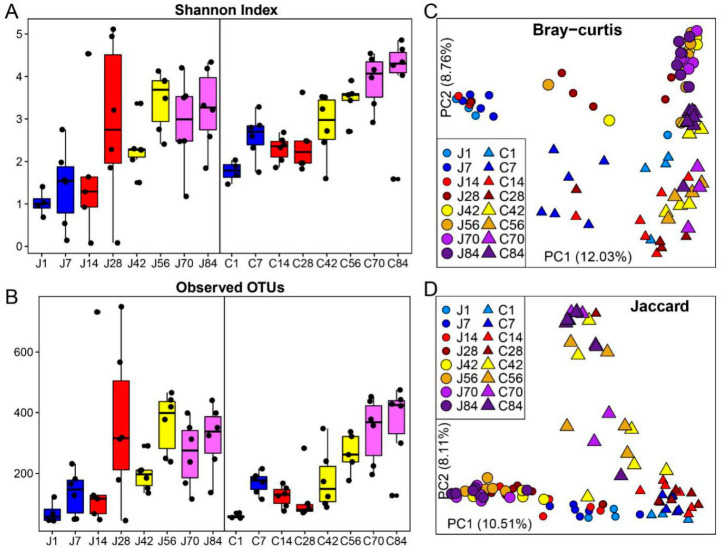
Microbial diversities and structure of the jejunum and colon at different ages in goat kids. (**A**,**B**): Longitudinal changes in the gut microbial community based on the Shannon Index and observed operational taxonomic units (OTUs). (**C**,**D**): The principal coordinate analysis (PCoA) based on the Bray–Curtis and Jaccard distances. Each point in (**A**,**B**) represents a unique sample. The growth phases of colostrum (d1 and d7), breast milk (d14 and d28), mixed feeding (d42 and d56) and starter feeding stages (d70 and d84) were differentiated by color (blue, red, yellow, and purple). Circles represent colon samples and triangles represent jejunum samples. J1 = jejunum d1; C1 = colon d1; the rest can be deduced by analogy.

Significant increases in microbial diversity, including the number of Observed OTUs (Figure 2A) and the Shannon Index (Figure 2B), were observed in both the jejunum and colon. In the jejunum, both bacterial diversity and richness on d1 were lower compared to d42–d84 (*p* < 0.05, Table S2). The community on d7 showed significantly low alpha diversities compared to d56, d70 and d84 (*p* < 0.05). Although the diversity increased with age from d14 to d84, the Observed OTUs and Shannon Index were not statistically significant (*p* > 0.05), except for a low Shannon Index on d42 compared with d56. In the colon, the lowest gut microbial diversity was observed on d1 (*p* < 0.05). Before supplementation starter on d30, both the Observed OTUs and Shannon Index on d 7, d14 and d28 were less diverse compared to d56, d70 and d84. On d42, colon bacteria showed lower alpha diversities compared with d70 and d84 (*p* < 0.05). Moreover, the diversity of biogeography of the gut microbiota was compared. The colon bacterial community had a high Shannon Index on d1 and d7 when compared to the jejunum at the same age (*p* < 0.05), however, other days were not significantly different (*p* > 0.05) (Table S3). Richness between the jejunum and colon was not significant across all sampling timepoints in this study (*p* > 0.05).

Regarding beta diversity, significant shifts in community membership and structure from d1 to d84 were observed on the principal coordinate analysis (PCoA)-based Bray–Curtis (Figure 2C) and Jaccard (Figure 2D) distances. Distinct clustering before- (d1–d28) and after-supplementation (d42–d84) in community membership and structure were also observed in both the jejunum and colon communities (Tables S4 and S5). The growth stage significantly influenced the gut microbial structure, for example, d1 and d7 showed distinct clustering with other ages based on Bray–Curtis distance. Moreover, distinct microbial beta diversity between the jejunum and colon at the same age was found.

### 3.2. The Dynamics of Gut Microbial Communities

We next examined how the core microbiota in the small and large intestines shifted from d1 to d84. At the phylum level (Figure S1), *Firmicutes*, *Bacteroidetes* and *Proteobacteria* represented the top three highest abundances across all samples. In the jejunum, although *Firmicutes* was always the most abundant phylum from d1 to d84, its abundance gradually deceased (d1–d84: 73.73%, 90.79%, 88.34%, 80.51%, 61.21%, 63.73%, 54.48%, 42.05%). However, *Bacteroidetes* and *Actinobacteria* increased in abundance with age (d1–d84: 1.03%, 3.11%, 4.46%, 8.00%, 1.07%, 6.98%, 6.85%, 13.20%; d1–d84: 0.14%, 0.21%, 0.65%, 1.07%, 20.58%, 2.99%, 21.88%, 24.48%). A resident bacterium (phylum *Proteobacteria*) in young goats existed in all ages and abundance was not observed to highly differentiate. Notably, some phyla changes exhibited remarkable stage characteristics. For example, after supplementation starter (d60), *Fusobacteria* and *Candidatus Saccharibacteria* appeared to increase first followed by a decrease. In colon contents, *Firmicutes*, *Bacteroidetes* and *Proteobacteria* were major phyla across all ages. While the abundance of *Proteobacteria* sharply decreased with age (d1–d84: 39.90%, 6.90%, 7.63%, 7.10%, 18.12%, 13.25%, 7.23%, 4.31%), *Bacteroidetes* rose with increasing age (d1–d84:19.66%, 33.14%, 54.97%, 61.70%, 34.44%, 37.93%, 45.01%, 51.69%). *Verrucomicrobia* and *Spirochaetes* appeared and increased after starter supplementation. Moreover, differences of the major phyla between the jejunum and colon were observed from d1 to d84.

**Figure 3:**
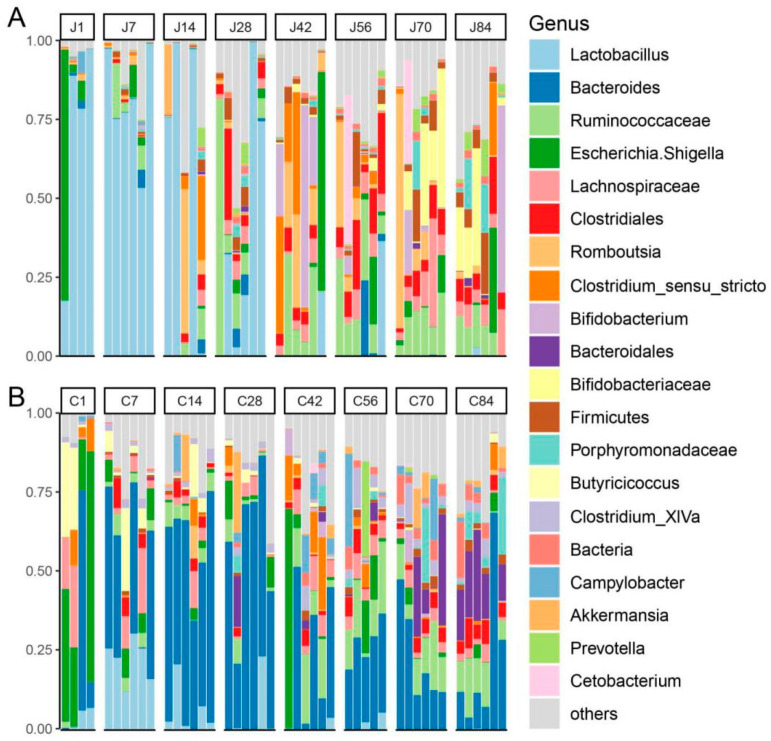
Microbial composition at the genus level. Stacked bar charts demonstrate temporal changes of the top 20 common genera in the jejunum (**A**) and colon (**B**) of goat kids. Each column represents one sample. Both the jejunum and colon share the same figure legend.

At the genus level, consistent genus changes with phyla were observed. In the jejunum (Figure 3A), the genus *Lactobacillus* belonging to Firmicutes decreased with age, especially after d28 (d1–d84: 70.55%, 80.60%, 54.63%, 38.09%, 3.50%, 6.18%, 0.04%, 0.51%), while the genera unclassified *Ruminococcaceae* (d1–d84: 0.02%, 4.70%, 2.86%, 18.59%, 12.24%, 15.62%, 12.33%, 8.74%), unclassified *Lachnospiraceae* (d1–d84: 0.03%, 0.10%, 1.14%, 3.42%, 3.33%, 5.77%, 10.54%, 6.86%) and unclassified *Clostridiales* (d1–d84: 0.01%, 0.42%, 1.63%, 7.15%, 3.96%, 13.12%, 5.89%, 7.09%) depicted an opposite pattern. The genera including *Bifidobacterium* and unclassified *Bifidobacteriaceae* increased in abundance between d42 and d84 (d1–d84: 0.08%, 0.01%, 0.11%, 0.13%, 18.13%, 1.10%, 3.81%, 9.86%; d1–d84: 0.01%, 0.00%, 0.01%, 0.08%, 0.72%, 1.13%, 17.52%, 12.73%). In the colon (Figure 3B), the genera of *Lactobacillus* (d1–d84: 3.58%, 22.28%, 5.79%, 4.13%, 0.75%, 1.63%, 0.01%, 0.10%) and *Butyricicoccus* (d1–d84: 14.20%, 7.17%, 3.78%, 1.34%, 0.57%, 0.40%, 0.08%, 0.49%) decreased with age. However, the unclassified *Ruminococcaceae* (d1–d84: 0.51%, 3.89%, 4.56%, 3.23%, 10.50%, 13.32%, 11.94%, 10.04%), unclassified *Clostridiales* (d1–d84: 0.02%, 3.49%, 2.69%, 0.67%, 2.14%, 3.87%, 3.70%, 5.83%) and *Campylobacter* (d1–d84: 0.62%, 0.00%, 1.93%, 0.53%, 4.84%, 6.10%, 3.99%, 0.67%) increased abundance after starter supplementation (d30). The genera including *Bacteroides*, unclassified *Lachnospiraceae* and *Clostridium_XlVa* were shown to be resident microbiota. Moreover, considering microbial biogeography, dominant genera in the jejunum shifted from *Lactobacillus* to unclassified *Ruminococcaceae*, unclassified *Lachnospiraceae* and unclassified *Clostridiales* through starter supplementation, whereas colon-dominant genera, except resident bacteria (e.g., *Bacteroides*, unclassified *Lachnospiraceae* and *Clostridium_XlVa*), shifted from genus *Lactobacillus* and *Butyricicoccus*, within d1–d28, to unclassified *Ruminococcaceae*, unclassified *Clostridiales* and *Campylobacter* after solid diet supplementation. Additionally, one specific staged genus, *Escherichia/Shigella*, enriched on d1 (jejunum: 22.10%; colon: 39.14%) and d42 (jejunum: 11.84%; colon: 12.66%) but significantly less in the other stages.

**Figure 4:**
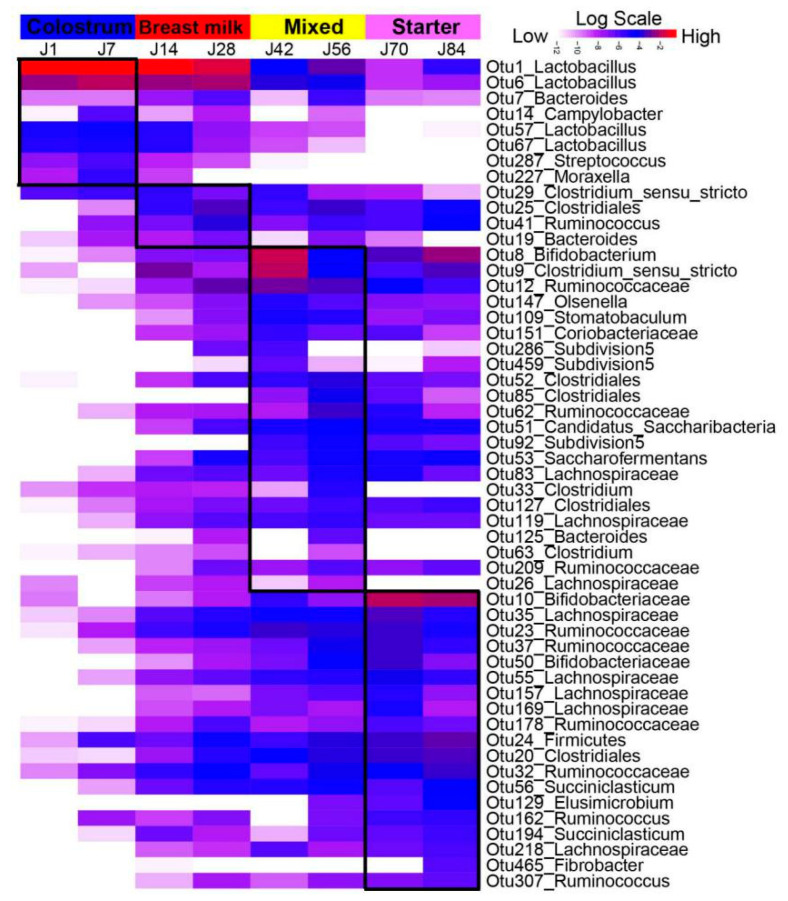
The jejunum stage-associated bacteria identified by linear discriminant analysis (LDA) effect size (LEfSe) analysis. The heat map shows the average relative abundances of ages on a log scale. The Log-scaled relative abundance heatmap of stage-related OTUs screened by Lefse (LDA > 2), in the jejunum microflora. The color of cells from white to red corresponds to the relative abundance of OTUs from low to high. The growth phases of colostrum (d1 and d7), breast milk (d14 and d28), mixed feeding (d42 and d56) and starter feeding stages (d70 and d84) were labeled with different colors. J1 = jejunum d1; others can be deduced by analogy.

To better understand the microbial alteration with age, growth stage-associated microbiota in the jejunum and colon were identified, respectively, by using LEfSe at the OTUs level. In jejunum (Figure 4), the OTUs associated with *Lactobacillus* (OTU1, OTU6, OTU57, OTU67) were identified as signatures for d1 and d7, while OUT1 and OTU6 still showed high abundances until d84. Other species including *Campylobacter* (OTU14), *Streptococcus* (OTU287) and *Moraxella* (OTU227) were enriched on d7. On d14 and d28, the stage-associated bacteria were *Clostridium sensu stricto* (OTU29), *Clostridiales* (OTU25), *Ruminococcus* (OTU41) and *Bacteroides* (OTU19). After starter supplementation on d30, *Bifidobacterium* (OTU8), *Clostridium sensu stricto* (OTU9), *Ruminococcaceae* (OTU12 and OTU62), *Subdivision5* (OTU92, OTU286, OTU459), *Clostridiales* (OTU52, OTU85, OTU127), *Lachnospiraceae* (OTU83, OTU119), and *Clostridium* (OTU33) had high relative abundances on d42 and d56. Moreover, these bacteria were abundant on adjacent ages (d28 and d70). During the starter phase (d70 and d84), enriched microbiota included *Bifidobacteriaceae* (OTU10, OTU50), *Lachnospiraceae* (OTU35, OTU55, OTU157, OTU169, OTU218,) *Ruminococcaceae* (OTU23, OTU37, OTU178, OTU32), *Ruminococcus* (OTU162, OTU307), *Clostridiales* (OTU20) and *Succiniclasticum* (OTU56, OTU194). Most of these signatures were abundant after d30.

**Figure 5:**
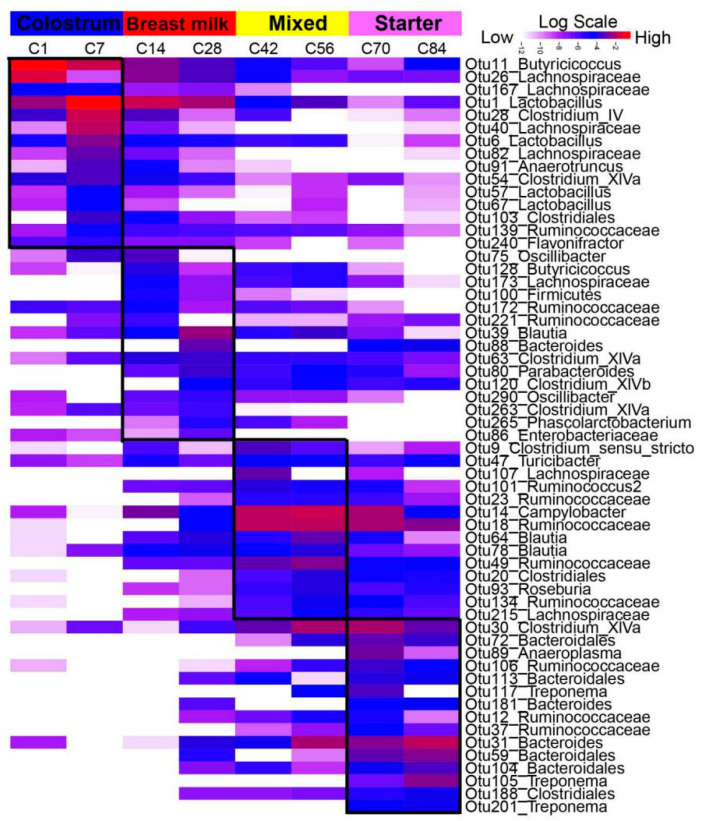
The colon stage-associated bacteria identified by LEfSe analyses. Heat map shows the average relative abundances of ages on a log scale. OTUs in this graph were statistically significant (*p* < 0.05) and had an LDA Score > 2, which was considered a significant effect size. The Log-scaled relative abundance heatmap of stage-related OTUs screened in colon contents. The color of cells from white to red corresponds to the relative abundance of OTUs from low to high. The growth phases of colostrum (d1 and d7), breast milk (d14 and d28), mixed feeding (d42 and d56) and starter feeding stages (d70 and d84) were labeled with different colors. C1 = colon d1, the rest can be deduced by analogy.

In the colon, LEfSe was also performed to identify the growth stage-associated microbiota (Figure 5). During the colostrum phase, the signature bacteria from d1 included *Butyricicoccus* (OTU11) and *Lachnospiraceae* (OTU26, OTU167), while d7-associated bacteria were *Lactobacillus* (OTU1, OTU6, OTU57, OTU67), *Lachnospiraceae* (OTU40, OTU82), *Clostridium IV* (OTU28), *Clostridium XlVa* (OTU54), *Clostridiales* (OTU103) and *Ruminococcaceae* (OTU139). Multiple signatures for d1 and d7 were abundant until d84, such as OTU1, OTU6 and OTU11. On d14, *Oscillibacter* (OTU75), *Butyricicoccus* (OTU128), *Lachnospiraceae* (OTU173), *Ruminococcaceae* (OTU172, OTU221) and *Blautia* (OTU39) were stage-associated bacteria, however, the stage-associated bacteria from d28 were *Bacteroides* (OTU88), *Clostridium XlVa* (OTU63, OTU263), *Clostridium XlVb* (OTU120), *Parabacteroides* (OTU80), and *Enterobacteriaceae* (OTU86). Regarding d42 and d56, *Clostridium sensu stricto* (OTU9), *Turicibacter* (OTU47), *Ruminococcaceae* (OTU23, OTU18, OTU49, OTU134), *Campylobacter* (OTU14), *Lachnospiraceae* (OTU107, OTU215), *Ruminococcus2* (OTU101), *Blautia* (OTU64, OTU78), *Clostridiales* (OTU20) and *Roseburia* (OTU93) showed a high abundance until d70. During the starter phase (d70 and d84), *Clostridium XlVa* (OTU30), *Clostridiales* (OTU188), *Bacteroidales* (OTU72, OTU113, OTU59, OTU104), *Bacteroides* (OTU181, OTU31), *Ruminococcaceae* (OTU106, OTU12, OTU37), *Treponema* (OTU117, OTU105, OTU201), and *Anaeroplasma* (OTU89) were found as the signature OTUs.

### 3.3. Network Analysis

**Figure 6:**
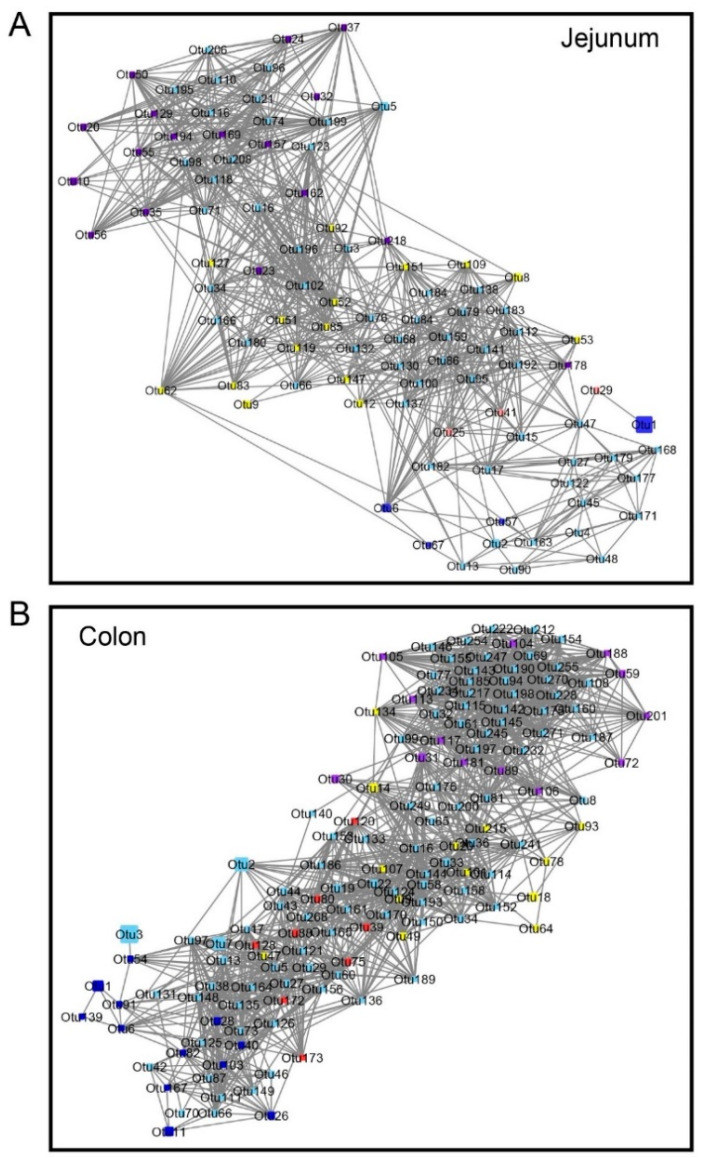
Network analysis. The FASTLSA algorithm was performed to identify the gut bacterial interactions with each age in the jejunum (**A**) and colon (**B**). LSA values greater than 0.6 or less than −0.6 were identified as the cutoff values. The size of the squares (OTUs) represents the relative abundances of OTUs temporally. The stage-associated bacteria identified by LEfSe were labeled with different colors: d1–d7: blue; d14–d28: red; d42–d56 yellow; d70–d84 purple; others were light blue color.

FAST LSA was performed in the jejunum and colon respectively, to capture the bacterial interactions in time series datasets with millions of co-variate time series. In both the jejunum and colon, the stage-associated bacterial signatures were also observed in a network analysis (Figure 6). In the jejunum (Figure 6A), the bacterial interactions were associated with growth stage, and more connections can be found within each stage. The stage-associated signatures showed a strong correlation with species in the corresponding growth stage. The hub OTUs that connected the two growth stages were observed as, for example, *Lactobacillus* (OTU6), *Clostridiales* (OTU25) and *Ruminococcus* (OTU41) linking the colostrum and breast milk phases and *Ruminococcaceae* (OTU23) connecting the mixed feeding and starter phases. A similar pattern was also observed in the colon (Figure 6B). The microbial interactions shifted with age. The hub OTUs identified as growth stage-associated bacteria included *Clostridium sensu stricto* (OTU9), *Campylobacter* (OTU14) and *Turicibacter* (OTU47). Additionally, more bacterial connections were found in the colon compared to the jejunum.

### 3.4. The Biogeography of Gut Microbiota

To better understand the signature bacteria for the small and large intestines, we also performed LEfSe for each growth stage, respectively. Across all ages, the top OTUs were repeatedly identified as intestinal, compartment-associated signatures. For example, *Romboutsia* (OTU5), *Clostridium sensu* strict (OTU9), *Ruminococcaoute* (OTU12), and *Clostridiales* (OTU20) enriched in the jejunum, whereas the colon had high relative abundances of *Bacteroides* (OTU4 and OUT7), *Butyricicoccus* (OTU11) and *Ruminococcaceae* (OTU18). During the colostrum phase (d1 and d7), the jejunum-associated bacteria included *Lactobacillus* (OTU1, OTU6) and OTU5 while the stage-associated taxa including *Butyricicoccus* (OTU11), *Escherichia/Shigella* (OTU2), *Clostridium XlVa* (OTU54), *Lachnospiraceae* (OTU82), *Anaerotruncus* (OTU91) and *Clostridiales* (OTU103) were also identified for signatures of the colon (Figure S2). From d14 to d28, the stage-associated bacteria including *Clostridiales* (OTU25) and *Ruminococcus* (OTU41) were identified as biomarkers for the jejunum, while stage-associated taxa in the colon also had high abundances compared to the jejunum, such as *Blautia* (OTU39), *Clostridium_XlVa* (OTU63), *Butyricicoccus* (OTU128), and *Ruminococcaceae* (OTU172) (Figure S3). When goats consumed breast milk plus starter on d42 and d56, the jejunum stage-associated bacteria including *Clostridium sensu stricto* (OTU9), *Ruminococcaceae* (OTU12, OTU62), *Lachnospiraceae* (OTU83, OTU119), *Clostridiales* (OTU127) and *Stomatobaculum* (OTU109) were identified, while the colon stage-associated taxa including *Ruminococcaceae* (OTU49, OTU134, OTU18), *Lachnospiraceae* (OTU215) and *Ruminococcus2* (OTU101) were also identified (Figure S4). On d70 and d84, most of the stage-associated bacteria in the jejunum including *Bifidobacteriaceae* (OTU10, OTU50), *Ruminococcaceae* (OTU23, OTU37, OTU178, OTU32), *Ruminococcus* (OTU162), *Clostridiales* (OTU20), *Lachnospiraceae* (OTU35, OTU55, OTU157, OTU169, OTU218), and *Succiniclasticum* (OTU56) were identified as biogeographic signatures, while stage-associated OTUs in the colon including *Clostridium_XlVa* (OTU30), *Clostridiales* (OTU188), *Bacteroidales* (OTU72, OTU113, OTU59, OTU104), *Bacteroides* (OTU181, OTU31), *Treponema* (OTU105, OTU201), and *Anaeroplasma* (OTU89) were also identified (Figure S5).

**Figure 7:**
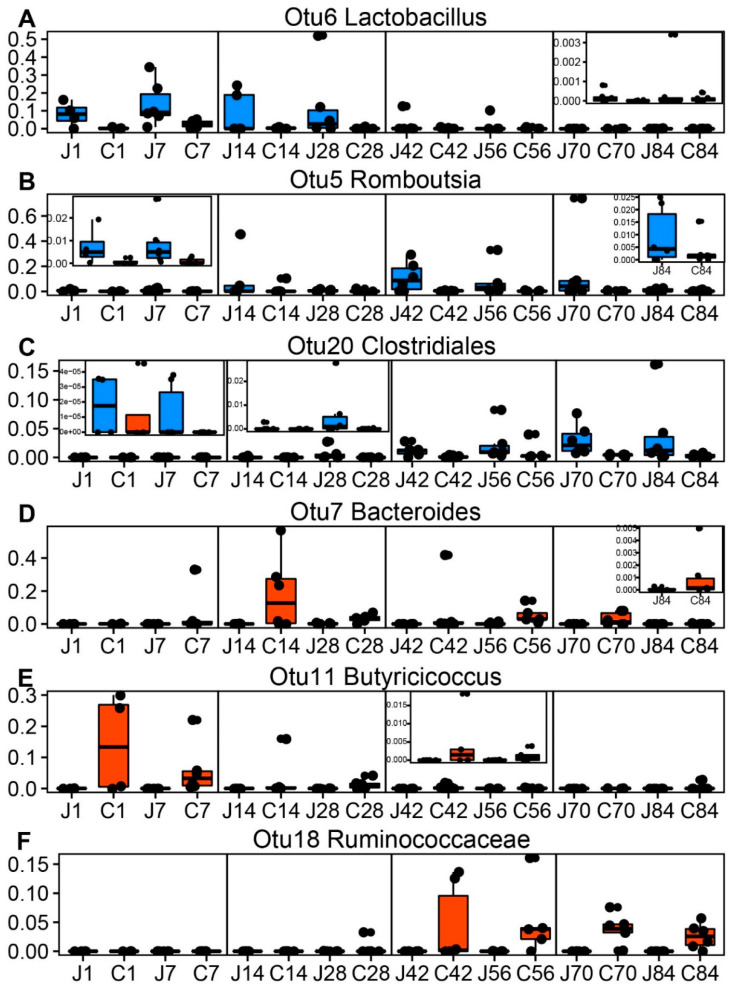
The boxplots of representative bacteria identified by LEfSe to display the microbial differences of the biogeography. The shared OTUs between the jejunum and colon including (**A**) *Lactobacillus* (OTU6), (**B**) *Romboutsia* (OTU5), (**C**) *Clostridiales* (OTU20), (**D**) *Bacteroides* (OTU7), (**E**) *Butyricicoccus* (OTU11) and (**F**) *Ruminococcaceae* (OTU18) represented the biogeographic differences. The dodgerblue and orangered colors represent the jejunum and colon, respectively. J1 = jejunum d1; C1 = colon d1; the rest can be deduced by analogy.

Next, the number of shared OTUs between the jejunum and colon were determined (Figure S6). Before supplementation starter, the shared number of OTUs increased. However, during the ages of d42 to d84, the shared number of OTUs was found consistent. To more deeply understand the signature microbiota that developed with spatial and temporal shifts, several dominant OTUs were displayed (Figure 7). *Lactobacillus* (OTU6) was enriched further in the jejunum than in the colon during d1–d28, and was less enriched after starter supplementation (d30) (Figure 7A). Two other OTUs, *Romboutsia* (OTU5) and *Clostridiales* (OTU20), appeared in all ages and were found to be jejunum signatures (Figure 7B,C). The colonic signatures including *Bacteroides* (OTU7), *Butyricicoccus* (OTU11) and *Ruminococcaceae* (OTU18) also showed association with age (Figure 7D–F). For example, OTU7 increased in abundance after d7, OTU11 decreased in abundance with increasing age, and OTU18 had a high relative abundance after d30.

### 3.5. Gut Microbiota Is Associated with Growth Performances

**Figure 8:**
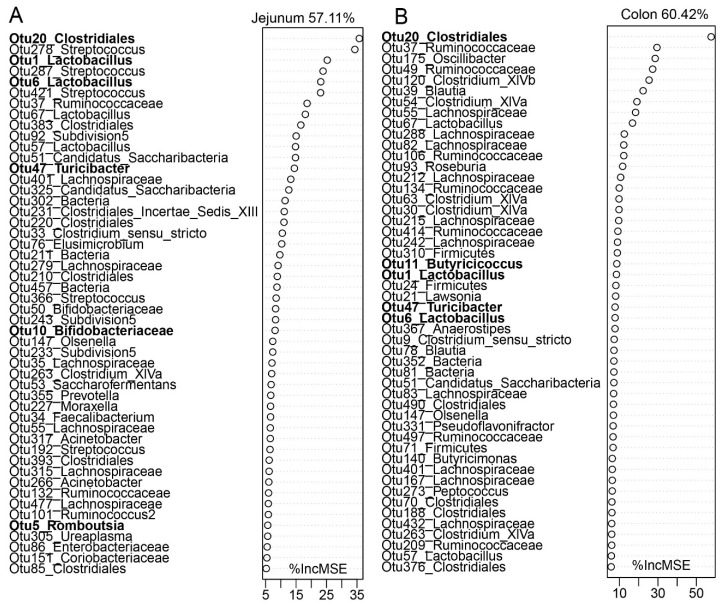
Growth performance-related bacteria identified by a random forest regression model. The top 50 growth-related (body weight) signature microbiota of the jejunum (**A**) and colon (**B**) in goat kids were listed based on the mean square error of predictions (%IncMSE). The bolded features denote potential probiotics or important commensal microbiota.

We next sought to identify the association between growth performance and gut microbiota. Firstly, we examined the correlation between body weight (BW) and alpha diversities of the gut microbiota (Figure S7). The alpha diversities in both the jejunum and colon showed a moderate, linear correlation with body weight. However, the Shannon Index and Observed OTUs in the colon had a stronger correlation (*r* = 0.54, *p* = 0.00013; r = 0.60, *p* = 0.000015) than those in the jejunum (*r* = 0.42, *p* = 0.0037; *r* = 0.39, *p* = 0.0079). Then, regression-based Random Forest using BW as the outcome and the top 500 bacterial features as predictors was performed (Figure 8). These features included members of both the core and stage-specific microbiota. For example, *Lactobacillus* (OTU1, OTU6, OTU57, OTU67) were found in both the jejunum and colon. Other stage-associated taxa including *Bifidobacteriaceae* (OTU10), *Ruminococcaceae* (OTU37), *Lachnospiraceae* (OTU55, OTU35), *Candidatus Saccharibacteria* (OTU51), *Olsenella* (OTU147), *Bifidobacteriaceae* (OTU50), *Subdivision5* (OTU92) and *Streptococcus* (OUT278) in the jejunum (Figure 8A) were positively correlated with BW. In the colon (Figure 8B), *Clostridium sensu stricto* (OTU9), *Clostridiales* (OTU20), *Turicibacter* (OTU47), *Ruminococcaceae* (OTU37, OTU106), *Clostridium XlVa* (OTU30) and *Roseburia* (OTU93) were positively correlated with body weight.

## 4. Discussion

### 4.1. Diversity of the Gut Community

This study investigated the temporally associated, dynamic changes of the microbial community in the jejunum and colon of goat kids from the undeveloped rumen phase to the rumination phase. In both sampling sites, the diversity and richness of bacterial communities increased with age, which is in accordance with results from previous research [7,16,17]. These results indicated that the microbial communities in both the small and large intestines gradually reached a mature and balanced state with increasing age. Supplementation with starter is another main factor that influenced the gut microbiota. The differences in gut microbial diversities before- and after-supplementation of starter were found to be significant in our results, which was also reported in a study related to the jejunum and colon microbiota in goats [7]. Liu et al. [5] observed that feeding starter supplementation increased the richness of colonic microbiota in newborn lambs. Similar results are also found in piglets [3]. Thus, supplementation of starter could be a co-factor influencing gut microbial development. Moreover, the structure of the microbiota in the gut, especially the colon, was affected by weaning at d60. When solid diet was the only nutrient source after weaning, the colon might increase its ability to ferment the diet for absorption of more nutrients to meet body requirements. Weaning generated enormous stress on the goat kids such as decreased appetite, diarrhea and even death, since it disrupts mother–child coexistence, the loss of passive immunity and the end of maternal microbial interference contributed to intestinal microbial disorders to various degrees [18,19]. Significant differences of rumen microbial diversities before- and after-weaning were also reported [20]. Therefore, factors such as age, solid diet supplementation and weaning demonstrate significant impacts on the gut microbiota.

### 4.2. Temporal Dynamics of the Core Microbiota

This study characterized the gut microbial composition at different growth stages to help answer various questions including (1) What is the core gut microbiota at each growth stage? (2) Which bacteria are early colonizers that impact later microbial composition? (3) Which bacteria are passengers, present only at a certain point in time due to diet or other factors?

Several OTUs classified as *Lactobacillus* in the gut, especially in the jejunum, were identified as signature bacteria for the colostrum phase and had high abundances before starter supplementation. Similar results with high abundance of *Lactobacillus* in the jejunum of newborn calves have been reported by Dias and their colleagues [9]. The maternal bacteria play important roles in the early colonization of the gut microbiota. Previous studies confirmed that the early jejunum microbes in suckled lambs were mainly derived from the mother’s teats and vagina [1], and that *Lactobacillus spp.* were prevalent in ewe vaginal microbial communities [21]. Colostrum is yet another factor influencing the microbiota in newborn goats. Malmuthuge et al. [22] suggested that timed feeding of high quality colostrum has a direct effect on bacterial colonization of the bovine small intestine, in particular, the mucosa-attached community that is in close contact with the host mucosal immune system. Another study reported that calves fed colostrum at 12 h after birth tended to decrease in *Lactobacillus* abundance associated with the colon mucosa compared to calves fed colostrum immediately after birth [23]. The abundant population of immunoglobulin (IgG, IgA and IgM) in colostrum also established the passive immunity of the infant [24], which created a relatively stable gut environment for the colonization of the microbiota. The nutrients in breast milk, such as amino acids, fats, oligosaccharides and glycans, could effectively promote the secretion of digestive enzymes in the intestine [25,26]. Polysaccharides and oligosaccharides in breast milk as prebiotics could inhibit the attachment of harmful bacteria by stimulating *Lactobacillus* growth. In addition, lactic acid, a metabolite of *Lactobacillus*, has also been confirmed to protect the intestine and suppress inflammation [27,28]. In this study, OTU1 and OTU6 (NCBI blast: *L. amylovorus*, *L. reuteri*) were found as colostrum-signatures and appeared until d84 in spite of decreased abundances with growth stages. *L. amylovorus* is known to contain functions related to the inhibition of intestinal inflammation as well as energy metabolism [29,30]. Direct supplementation or prebiotic modulation of *L. reuteri* may be an attractive preventive and/or therapeutic avenue against inflammatory diseases [31]. Therefore, it is proposed that OTU1 and OTU6 could be used as potential probiotics for goat kids.

During the breast milk phase, the signature microbiota in the jejunum for d14 and d28 were *Clostridium sensu stricto* (OTU29), *Clostridiales* (OTU25), *Ruminococcus* (OTU41) and *Bacteroides* (OTU19). These signature microbiota persisted from a breast milk-based stage, d7, to the starter-based stage, d84, which indicates that they were normal residents in the jejunum. The genera *Clostridium sensu stricto*, *Ruminococcus* and *Bacteroides*, are well-known for their characteristic degradation of saccharides [7,9,32]. In the colon, the signature bacteria including *Ruminococcaceae* (OTU172, OTU221), *Bacteroides* (OTU88), *Clostridium XlVa* (OTU63, OTU263) and, *Clostridium XlVb* (OTU120) were identified. In both gut compartments, most of those signature bacteria were early colonizers and persisted throughout the entire study. It was assumed that these early colonized bacteria can shape gut microbial composition in later life and allow the host to digest solid diet (starter) more easily.

After starter supplementation on d30, *Clostridium perfringens* (OTU9), was classified as a signature for both the jejunum and colon on d42 and d56. The gut microbiota may increase its ability to resist stress from a solid diet. *C. perfringens* is a major cause of enteric diseases in sheep, goats, and other animals, since it produces several potent toxins in the intestines of the animal, some of which have proven to be involved in the pathogenesis of certain diseases [33]. Increases in *C. perfringens* abundance in the jejunum can be considered a gut distress from the intake of solid diet, which may lead to diarrhea or gut inflammation. Similar shifts in other bacteria were also observed in the colonic community. *Campylobacter hyointestinalis* (OTU14), found as a colonic signature in our results, was found in increasing abundance when it was isolated from patients with diarrhea [34]. Simultaneously, adjustments of gut microbial communities in response to a solid diet were observed. For example, the jejunum enriched *Bifidobacterium pseudolongum* (OTU8) that can reduce the susceptibility to oxidation of proteins in the colon and the small bowel, and thus, be considered as an influence or biomarker of oxidative damage [35]. *Turicibacter* (OTU47) is identified as the signature for the colon and was found significantly abundant until d84 in this study. *Turicibacter* is known to reduce the susceptibility to Salmonella infection [36]. Thus, *Turicibacter* (OTU47) plays some potentially positive roles in the goat microbial community and may, consequently, promote growth performance. Moreover, *Ruminococcus bromii* (OTU12, OTU23 and OTU62) increased relative abundances in both gut compartments. *R. bromii* is known to degrade certain forms of particulate resistant starch [37]. Therefore, the gut microbiota in this stage can alter in response to solid diet intake distress and increase respective bacteria to digest the associated plant materials.

On d70 and d84, starter was the only nutrient source for the goat kids. *Bifidobacterium bohemicum* (OTU10, OTU50) was found as a signature for the jejunum during this period, and persistently appeared in all ages. OTU10 was also positively correlated with body weight. *Bifidobacteria* are among the most prevalent gut commensals, playing crucial functional roles that start from their early colonization of the infant gastrointestinal tract and last throughout the animal’s life span [38]. One study confirmed that *Bifidobacterium* can degrade polysaccharides in the gut [39]. Thus, *B. bohemicum* is a main nutrient-degrader in the jejunum and can be considered as a potential probiotic. Moreover, *Lachnospiraceae* (OTU35, OTU55, OTU157, OTU169 and OTU218), which was enriched in the jejunum after starter supplementation and was also positively correlated with body weight, has the ability to degrade cellulose and hemicellulose components of plant material to short chain fatty acids for energy requirements of the host [40].

### 4.3. Biogeography Dissimilarities of Gut Microbiota

Investigation of the biogeography of the microbiota could provide more insights into the bacterial functions within each gut compartment. In this study, the dissimilarities between the jejunum and colon across all growth stages were investigated. Distinct structure and membership of the gut microbiota across all ages was observed between the jejunum and colon. Alpha diversity in the colon had higher values compared to the jejunum in the colostrum phase, but did not maintain this trend in later stages. Our results are similar with studies found across goats [7], piglets [41], calves [9], and murine [18]. This is due to the different physical and chemical properties of the jejunum and colon including pH, morphology, excreta, physical structure, function, etc. [42]. Notably, with increasing age, the developments of the jejunum and colon throughout differentiating environments results in associated differences of the inhabited microbial community structure and composition. This explanation is supported by the identification of multiple growth-associated signatures for biogeography. Additionally, there are still several bacteria that were consistent as biomarkers for the jejunum and colon. The jejunum is known as a main niche for food digestion and nutrient absorption [1], and naturally many signature bacteria related to nutrient metabolism were observed. *Romboutsia* (OTU5) had a high abundance in the jejunum across age compared to the colon. The genus *Romboutsia* associates with carbohydrate utilization, fermentation of amino acids and anaerobic respiration [43]. *Clostridiales* (OTU20), which had high abundances across all ages in the jejunum, is a saprophytic organism that ferments plant polysaccharides. Moreover, OTU5 and OTU20 portrayed a positive correlation with body weight, which indicated that the commensal bacteria related to nutrient metabolism can enhance animal performance. The colon, as a part of the large intestine, is the final stage of the digestive system. Compared with the jejunum, the signature bacteria for the colon included *Bacteroides* (OTU4 *B. fragilis* and OUT7 *B. acidifaciens*), *Butyricicoccus pullicaecorum* (OTU11) and *Ruminococcaceae* (OTU18). *B. fragilis* and *B. acidifaciens* are commensal bacteria in the colon. The latter was concluded as a potential agent for modulating metabolic disorders such as diabetes and obesity in mice [44]. *B. pullicaecorum* (OTU11) was found to be abundant in the colon from d0 to d56. As a butyrate-producer, *B. pullicaecorum* has anti-inflammatory effects for people suffering from inflammatory bowel disease and can be considered as a probiotic [45]. *Ruminococcaceae* can maintain gut health and degrade plant materials in a solid diet [40], which agrees with our results regarding OTU18 as an abundant organism after supplementation of a solid diet.

The local spatial organization of the gut microbiota influences various properties including colonization, metabolism, host–microbe and inter-microbial interactions, and community stability [46]. The chyme passes through the small intestine faster and stays in the large intestine for a longer time [47]. In this study, shared OTUs were observed between the jejunum and colon. The neutral model was performed to find their association; however, it failed due to too few shared OTUs (data not shown), which may be due to reaching anatomical distances between the jejunum and colon. In a previous study, several models including linear regression and logistic models also analyzed the biogeography of the gut microbiota in macaque and indicated a moderate correlation between the small intestine and stool samples [48]. Sheth et al. found robust associations between *Bacteroidales* in all gut compartments of the mouse and phylogenetically clustered local regions of the microbiota associated with dietary perturbation [46]. To our knowledge, there is no research to assess the spatial association of the gut microbiota in ruminants. Therefore, further investigation for the identification of the spatial dynamics for adjacent gut niches is needed.

## 5. Conclusions

In this study, we characterized the temporal dynamics of the microbiota in the jejunum and colon of neonatal goats from birth to postweaning. The gut microbiota matures with age. The signature gut microbiota for each age were identified. Corresponding to the growth stage, the signature microbiota in both compartments shift temporally, which is associated with diet changes, gut anatomical development, and microbial interactions. Early microbial colonization can influence gut microbiota postweaning. Moreover, spatial differences of the gut microbiota were observed. The colon had a more complex bacterial community than the jejunum across all growth stages. Based on the correlation with body weight, several microbial colonizers can be considered as potential probiotics, which provides insights that improving the gut microbiota can enhance animal health and production.

## Supplementary Materials

The following are available online at https://www.mdpi.com/2076-2607/8/8/1111/s1, Table S1. Nutritional components of starter; Table S2. The statistical differences of alpha diversity of goat gut microbial communities at different ages; Table S3. The statistical differences of alpha diversity of goat gut microbial communities between the jejunum and colon; Table S4. Analysis of similarity (ANOSIM) test for determination of microbial dissimilarities at different ages based on both Bray–Curtis and Jaccard distances; Table S5. Analysis of similarity (ANOSIM) test for determination of microbioal dissimilarities between the jejunum and colon based on Bray–Curtis and Jaccard distances; Figure S1. Microbial composition at the phylum level. Figure S2. The identification of stage-associated bacteria in the jejunum and colon at the colostrum phase. Figure S3. The identification of stage-associated bacteria in the jejunum and colon at the breast milk phase. Figure S4. The identification of stage-associated bacteria in the jejunum and colon at the mixed feeding phase. Figure S5. The identification of stage-associated bacteria in the jejunum and colon at the starter phase. Figure S6. The number of shared OTUs between the jejunum and colon at different ages in goats. Figure S7. Spearman correlation between body weight (BW) and alpha diversity of the gut microbiota.
