# Supplementary material for: Longitudinal Investigation of the Gut Microbiota in Goat Kids from Birth to Postweaning
Source: Microorganisms. 2020 Jul 24;8(8):1111. doi: 10.3390/microorganisms8081111 (PMC7463816; doi:10.3390/microorganisms8081111)
Supplement: Supplementary file 1 [file microorganisms-08-01111-s001.pdf]

Supplementary Information

Longitudinal investigation of the gut microbiota in goat kids from birth to postweaning

Yimin Zhuang [1,†], Jianmin Chai [1,2,†], Kai Cui[1], Yanliang Bi[1], Qiyu Diao [1], Wenqin Huang [1], Hunter Usdrowski [2] and Naifeng Zhang [1,*]

[1] Feed Research Institute, Chinese Academy of Agricultural Sciences, Key Laboratory of Feed Biotechnology of the Ministry of Agriculture, Beijing 100081, China; zym1164323345@163.com (Y.Z.); jchai@uark.edu (J.C.); cuikai@caas.cn (K.C.); vetbi2008@163.com (Y.B.); diaoqiyu@caas.cn (Q.D.); m13121271017@163.com (W.H.); hmusdrow@uark.edu (H.U.)

[2] Department of Animal Science, Division of Agriculture, University of Arkansas, Fayetteville, AR 72701, USA

* Correspondence: zhangnaifeng@caas.cn, Tel.: +86(10)-8210-6055

†These authors contributed equally to this work

Table S1. Nutritional components of starter

Table S2. The statistical differences of alpha diversity of goat gut microbial communities at different ages

Table S3. The statistical differences of alpha diversity of goat gut microbial communities between the jejunum and colon

Table S4. Analysis of similarity (ANOSIM) test for determination of microbiota dissimilarities at different ages based on both Bray-Curtis and Jaccard distances

Table S5. Analysis of similarity (ANOSIM) test for determination of microbiota dissimilarities between the jejunum and colon based on both Bray-Curtis and Jaccard distances

Figure S1. Microbial composition at the phylum level.

Figure S2. The identification of stage-associated bacteria in the jejunum and colon at the colostrum phase.

Figure S3. The identification of stage-associated bacteria in the jejunum and colon at the breast milk phase.

Figure S4. The identification of stage-associated bacteria in the jejunum and colon at the mixed feeding phase.

Figure S5. The identification of stage-associated bacteria in the jejunum and colon at the starter phase.

Figure S6. The number of shared OTUs between the jejunum and colon at different ages of goats

Figure S7. Spearman correlation between body weight (BW) and alpha diversity of the gut microbiota

Table S1. Nutritional components of starter

Items	Starter Pellets
Chemical composition, % of DM basis	
DM (%)	95.25
Total Energy (MJ/Kg)	18.03
CP (%)	19.63
EE (%)	3.40
NDF (%)	29.62
ADF (%)	8.64
Ash (%)	7.99
Ca (%)	0.95
P (%)	0.70

DM: Dry matter; CP: Crude protein; EE: Ether extract; NDF: Neutral detergent fiber; ADF: Acid detergent fiber; Ash: Crude ash; Ca: Calcium; P: Phosphorus

Table S2. The statistical differences of alpha diversity of goat gut microbial communities at different ages

Items	location	Group1	Group2	Wilcoxon test *p-value*
Shannon Index	Jejunum	J1	J7	0.476
		J1	J14	0.73
		J1	J28	0.114
		J1	J42	0.01
		J1	J56	0.01
		J1	J70	0.019
		J1	J84	0.01
		J7	J14	0.931
		J7	J28	0.18
		J7	J42	0.132
		J7	J56	0.004
		J7	J70	0.065
		J7	J84	0.015
		J14	J28	0.178
		J14	J42	0.178
		J14	J56	0.126
		J14	J70	0.247
		J14	J84	0.126
		J28	J42	0.589
		J28	J56	0.589
		J28	J70	0.937
		J28	J84	0.818
		J42	J56	0.009
		J42	J70	0.132
		J42	J84	0.132
		J56	J70	0.589
		J56	J84	0.937
		J70	J84	0.699

Table S2. *Cont.*

Shannon Index	Colon	C1	C7	0.038
		C1	C14	0.067
		C1	C28	0.114
		C1	C42	0.067
		C1	C56	0.016
		C1	C70	0.01
		C1	C84	0.067
		C7	C14	0.31
		C7	C28	0.485
		C7	C42	0.589
		C7	C56	0.03
		C7	C70	0.004
		C7	C84	0.065
		C14	C28	0.818
		C14	C42	0.132
		C14	C56	0.004
		C14	C70	0.002
		C14	C84	0.065
		C28	C42	0.485
		C28	C56	0.052
		C28	C70	0.009
		C28	C84	0.065
		C42	C56	0.126
		C42	C70	0.041
		C42	C84	0.065
		C56	C70	0.247
		C56	C84	0.082
		C70	C84	0.485

Table S2. *Cont.*

Observed OTUs	Jejunum	J1	J7	0.114
		J1	J14	0.286
		J1	J28	0.067
		J1	J42	0.01
		J1	J56	0.01
		J1	J70	0.019
		J1	J84	0.01
		J7	J14	0.662
		J7	J28	0.132
		J7	J42	0.18
		J7	J56	0.002
		J7	J70	0.065
		J7	J84	0.015
		J14	J28	0.329
		J14	J42	0.126
		J14	J56	0.126
		J14	J70	0.247
		J14	J84	0.126
		J28	J42	0.24
		J28	J56	0.818
		J28	J70	0.699
		J28	J84	0.937
		J42	J56	0.009
		J42	J70	0.31
		J42	J84	0.065
		J56	J70	0.093
		J56	J84	0.394
		J70	J84	0.485

Table S2. *Cont.*

Observed OTUs	Colon	C1	C7	0.01
		C1	C14	0.01
		C1	C28	0.01
		C1	C42	0.01
		C1	C56	0.016
		C1	C70	0.01
		C1	C84	0.01
		C7	C14	0.065
		C7	C28	0.065
		C7	C42	0.818
		C7	C56	0.03
		C7	C70	0.004
		C7	C84	0.041
		C14	C28	0.18
		C14	C42	0.485
		C14	C56	0.004
		C14	C70	0.002
		C14	C84	0.015
		C28	C42	0.093
		C28	C56	0.03
		C28	C70	0.009
		C28	C84	0.004
		C42	C56	0.178
		C42	C70	0.026
		C42	C84	0.026
		C56	C70	0.247
		C56	C84	0.178
		C70	C84	0.699

Table S3. The statistical differences of alpha diversity of goat gut microbial communities between jejunum and colon

Items	Group1	Group2	Wilcoxon test *p-value*
Shannon Index	J1	C1	0.029
	J7	C7	0.026
	J14	C14	0.126
	J28	C28	0.699
	J42	C42	0.132
	J56	C56	0.931
	J70	C70	0.132
	J84	C84	0.18
Observed OTUs	J1	C1	0.886
	J7	C7	0.394
	J14	C14	0.537
	J28	C28	0.093
	J42	C42	0.485
	J56	C56	0.126
	J70	C70	0.24
	J84	C84	0.394

Table S4. Analysis of similarity (ANOSIM) test for determination microbiota dissimilarities at different ages based on the Bray-Curtis and Jaccard distances

Items	Location	Group1	Group2	*r*	Wilcoxon test *p-value*
Bray-Curtis	Jejunum	J1	J7	-0.063	0.59
		J1	J14	-0.044	0.545
		J1	J28	0.091	0.245
		J1	J42	0.647	0.003
		J1	J56	0.623	0.008
		J1	J70	0.857	0.004
		J1	J84	0.813	0.007
		J7	J14	-0.016	0.459
		J7	J28	0.3	0.019
		J7	J42	0.846	<0.001
		J7	J56	0.822	0.003
		J7	J70	0.985	0.004
		J7	J84	0.939	0.003
		J14	J28	-0.037	0.532
		J14	J42	0.343	0.063
		J14	J56	0.288	0.08
		J14	J70	0.549	0.002
		J14	J84	0.539	0.005
		J28	J42	0.446	0.008
		J28	J56	0.206	0.056
		J28	J70	0.489	0.003
		J28	J84	0.383	<0.001
		J42	J56	0.165	0.092
		J42	J70	0.172	0.1
		J42	J84	0.25	0.053
		J56	J70	-0.102	0.809
		J56	J84	0.091	0.124
		J70	J84	-0.113	0.888

Table S4. *Cont.*

Bray-Curtis	Colon	C1	C7	0.746	0.008
		C1	C14	0.492	0.022
		C1	C28	0.317	0.032
		C1	C42	0.294	0.068
		C1	C56	0.531	0.015
		C1	C70	0.794	0.008
		C1	C84	0.782	0.003
		C7	C14	0.274	0.029
		C7	C28	0.298	0.013
		C7	C42	0.726	0.002
		C7	C56	0.84	<0.001*
		C7	C70	0.854	0.003
		C7	C84	0.909	0.002
		C14	C28	0.019	0.356
		C14	C42	0.269	0.015
		C14	C56	0.333	0.014
		C14	C70	0.541	0.008
		C14	C84	0.789	0.002
		C28	C42	0.122	0.139
		C28	C56	0.117	0.176
		C28	C70	0.254	0.072
		C28	C84	0.441	0.029
		C42	C56	-0.179	0.963
		C42	C70	0.098	0.181
		C42	C84	0.272	0.037
		C56	C70	-0.064	0.611
		C56	C84	0.347	0.043
		C70	C84	-0.1	0.833

Table S4. *Cont.*

Jaccard	Jejunum	J1	J7	0.409	0.023
		J1	J14	0.088	0.273
		J1	J28	0.655	0.012
		J1	J42	0.948	0.003
		J1	J56	0.996	0.002
		J1	J70	0.996	0.004
		J1	J84	0.984	0.007
		J7	J14	0.083	0.215
		J7	J28	0.544	0.008
		J7	J42	0.887	<0.001
		J7	J56	0.933	0.003
		J7	J70	0.95	0.004
		J7	J84	0.907	0.003
		J14	J28	0.168	0.107
		J14	J42	0.464	0.014
		J14	J56	0.531	0.007
		J14	J70	0.581	0.006
		J14	J84	0.544	0.011
		J28	J42	0.261	0.01
		J28	J56	0.115	0.074
		J28	J70	0.335	0.003
		J28	J84	0.248	0.011
		J42	J56	0.111	0.103
		J42	J70	0.111	0.087
		J42	J84	0.23	0.025
		J56	J70	0.154	0.116
		J56	J84	0.07	0.21
		J70	J84	-0.022	0.498

Table S4. *Cont.*

Jaccard	Colon	C1	C7	0.901	0.008
		C1	C14	0.361	0.03
		C1	C28	0.381	0.026
		C1	C42	0.552	0.009
		C1	C56	0.944	0.007
		C1	C70	0.948	0.005
		C1	C84	0.897	0.003
		C7	C14	0.607	0.002
		C7	C28	0.713	0.004
		C7	C42	0.878	0.002
		C7	C56	0.981	<0.001
		C7	C70	0.993	0.003
		C7	C84	0.946	0.002
		C14	C28	0.144	0.068
		C14	C42	0.481	0.004
		C14	C56	0.827	0.002
		C14	C70	0.889	0.007
		C14	C84	0.896	0.002
		C28	C42	0.18	0.103
		C28	C56	0.461	0.016
		C28	C70	0.535	0.019
		C28	C84	0.613	0.007
		C42	C56	-0.013	0.47
		C42	C70	0.185	0.085
		C42	C84	0.278	0.033
		C56	C70	0.024	0.337
		C56	C84	0.293	0.049
		C70	C84	-0.097	0.951

Table S5. Analysis of similarity (ANOSIM) test for determination of microbiota dissimilarities between jejunum and colon on the Bray-Curtis and Jaccard distances

Items	Group1	Group2	*r*	Wilcoxon test *p-value*
Bray-Curtis	J1	C1	0.5	0.072
	J7	C7	0.844	0.004
	J14	C14	0.504	0.001
	J28	C28	0.533	0.001
	J42	C42	0.611	0.003
	J56	C56	0.725	0.001
	J70	C70	0.919	<0.001
	J84	C84	0.822	0.001
Jaccard	J1	C1	0.49	0.053
	J7	C7	0.985	0.004
	J14	C14	0.896	0.001
	J28	C28	0.626	0.001
	J42	C42	0.746	0.002
	J56	C56	0.92	0.001
	J70	C70	0.944	<0.001
	J84	C84	0.826	0.001

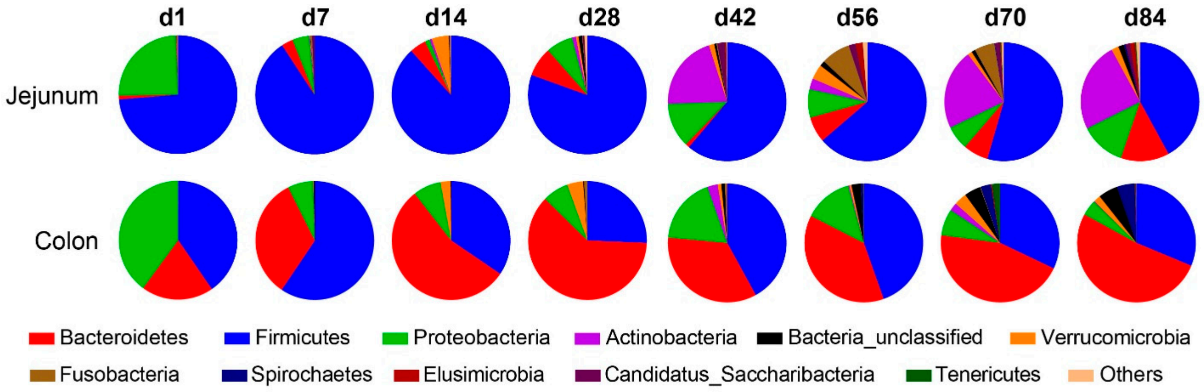

Figure S1. Microbial composition at the phylum level. The top and bottom panel of the pie chart portray the changes in phylum composition of the jejunum and colon at different ages in goat kids.

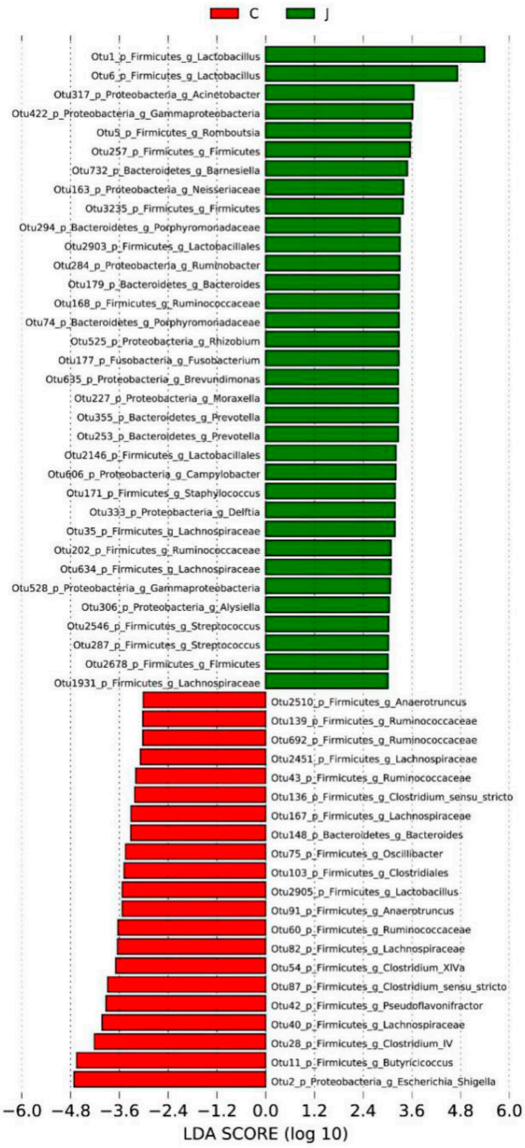

Figure S2. The identification of stage-associated bacteria in jejunum and colon at colostrum phase. OTUs in this graph were statistically significant ($p < 0.05$) and had an LDA Score >3, considered a significant effect size. J = jejunum, C= colon.

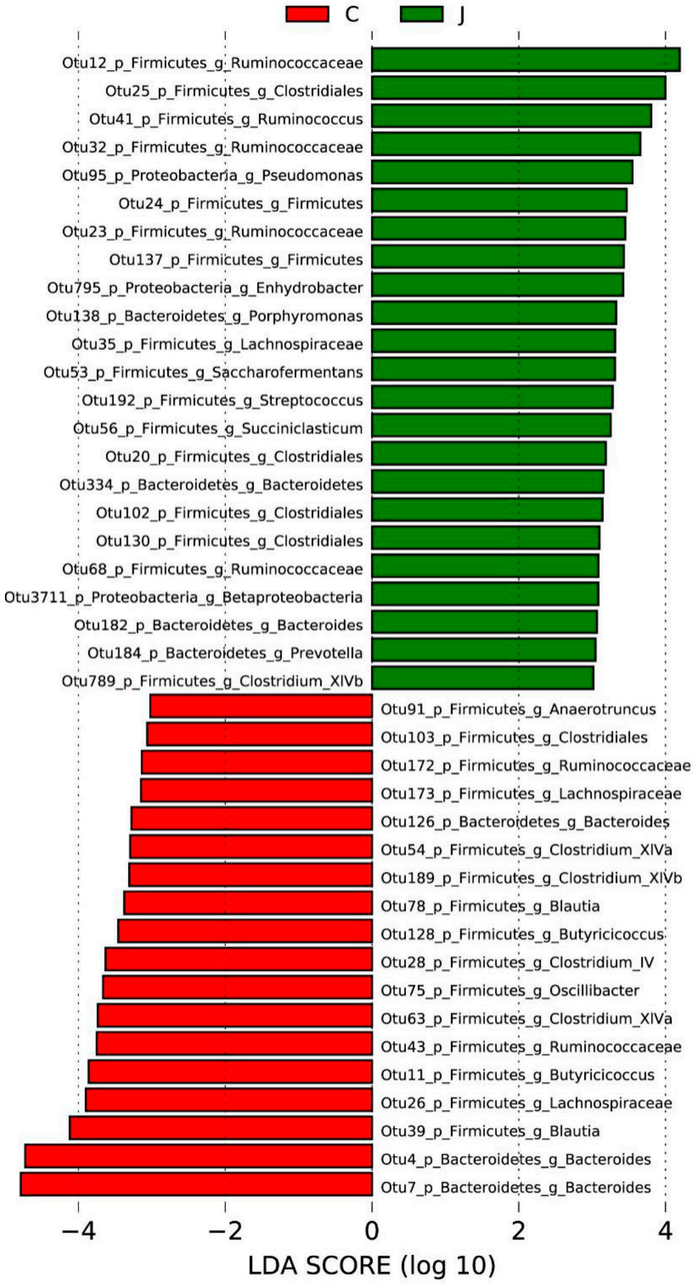

Figure S3. The identification of stage-associated bacteria in jejunum and colon at breast milk phase. OTUs in this graph were statistically significant ($p < 0.05$) and had an LDA Score >3, considered a significant effect size. J = jejunum, C= colon.

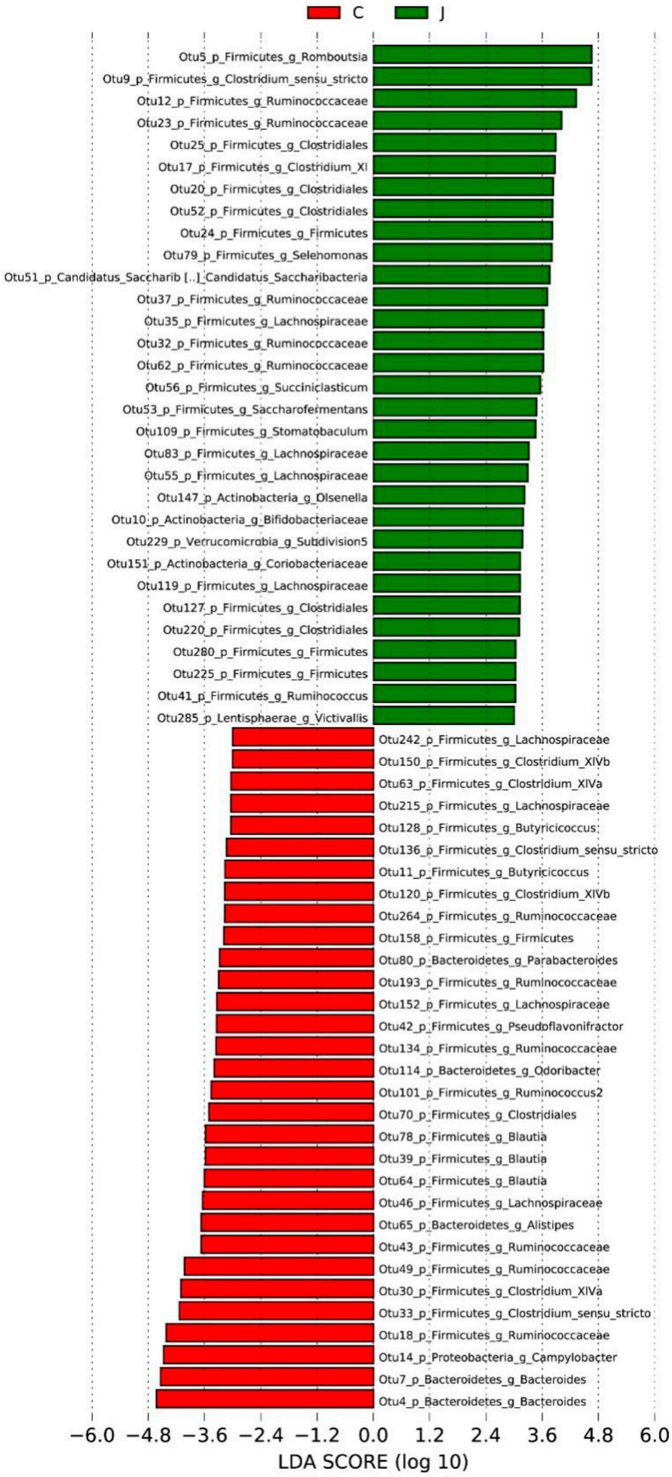

Figure S4. The identification of stage-associated bacteria in jejunum and colon at mixed feeding phase. OTUs in this graph were statistically significant (p < 0.05) and had an LDA Score >3, considered a significant effect size. J = jejunum; C= colon.

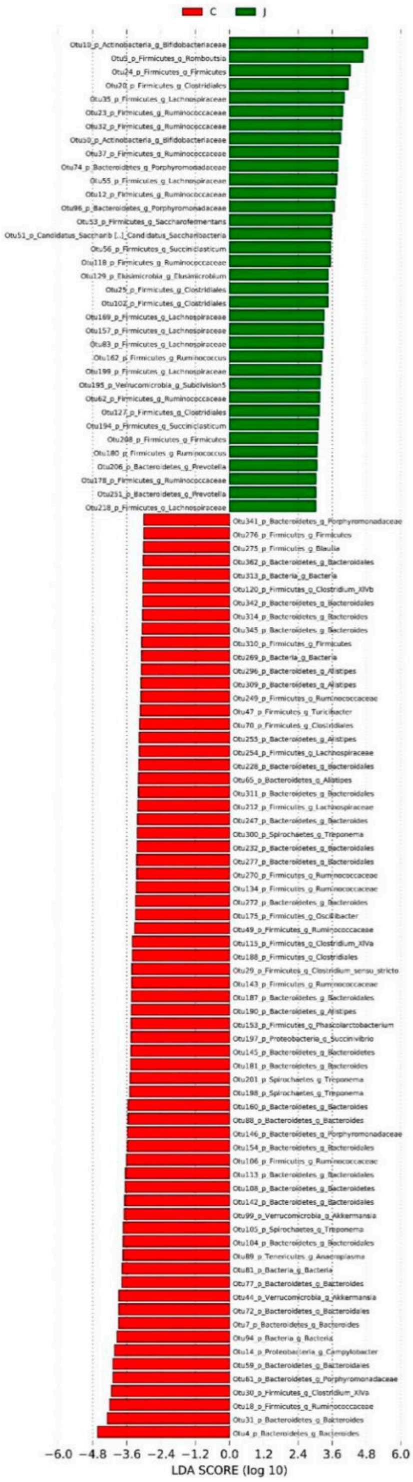

Figure S5. The identification of stage-associated bacteria in jejunum and colon at starter phase. OTUs in this graph were statistically significant ($p < 0.05$) and had an LDA Score >3, considered a significant effect size. J = jejunum, C= colon.

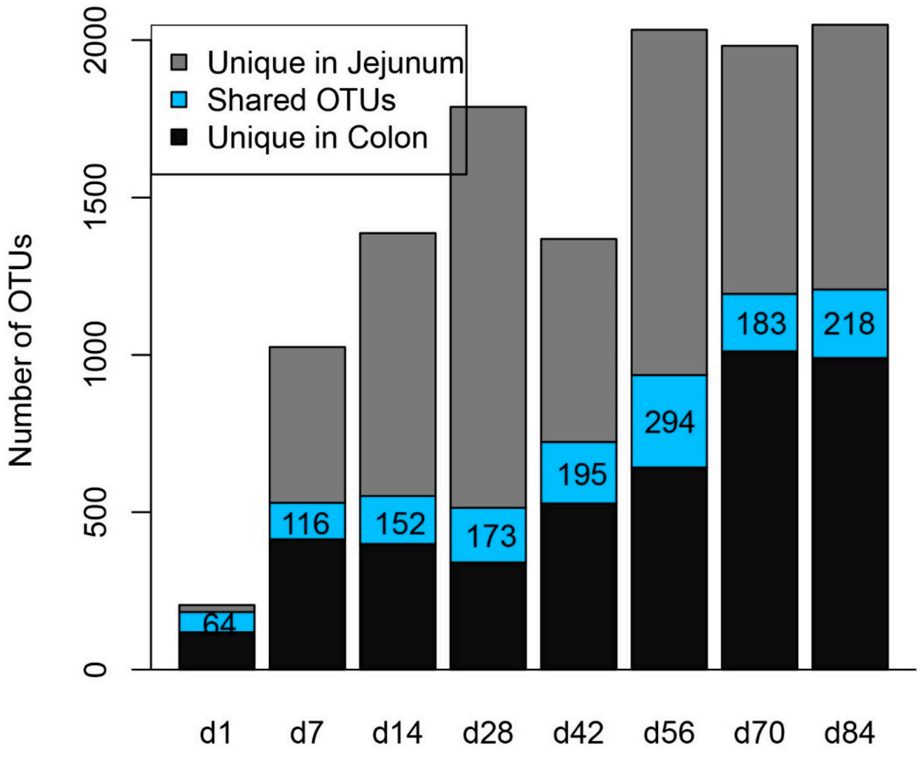

Figure S6. The number of shared OTUs between jejunum and colon at different ages of goats.

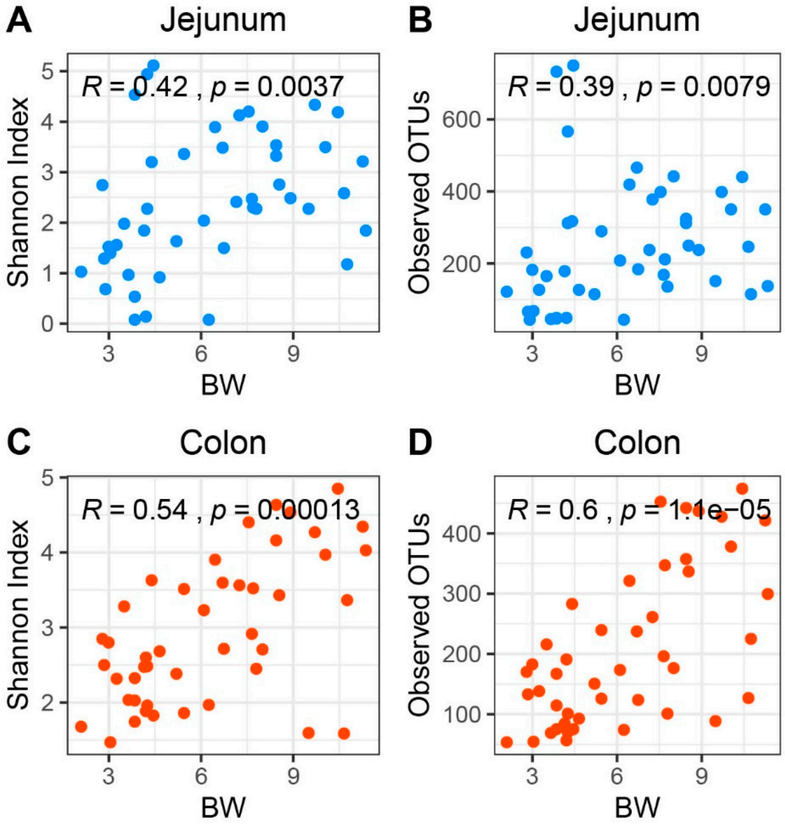

Figure S7. Spearman correlation between body weight (BW) and alpha diversity of gut microbiota. A: The correlation between the Shannon Index in the jejunum and body weight. B: The correlation between Observed OTUs in the jejunum and body weight. C: The correlation between the Shannon Index in the colon and body weight. D: The correlation between Observed OTUs in the jejunum and body weight. BW= body weight.
